# Pseudohypoparathyroidism: application of the Italian common healthcare-pathway for a homogeneous clinical approach and a shared follow up

**DOI:** 10.1186/s13052-021-01000-z

**Published:** 2021-03-04

**Authors:** Daniele Tessaris, Elisa Bonino, Giovanna Weber, Malgorzata Wasniewska, Domenico Corica, Marco Pitea, Giuseppe Scirè, Manuela Caruso-Nicoletti, Danilo Fintini, Luisa de Sanctis

**Affiliations:** 1 Department of Pediatric Endocrinology, Regina Margherita Children’s Hospital – Health and Science City, Turin, Italy; 2 Department of Public Health and Pediatrics, University of Turin, Piazza Polonia 94, 10126 Torino (TO), Italy; 3 Department of Pediatrics, San Raffaele Hospital, University of Milan, Milan, Italy; 4 Department of Pediatrics, Gynecological, Microbiological and Biomedical Sciences, University of Messina, Messina, Italy; 5 Endocrinology Unit, Pediatric University Department, “Bambino Gesù” Children’s Hospital-IRCCS, Rome, Italy; 6 Department of Pediatrics, University of Catania, Catania, Italy

**Keywords:** Pseudohypoparathyroidism, GNAS locus, *iPPSDs*, PHP, Healthcare pathway

## Abstract

**Background:**

Pseudohypoparathyroidism (PHP) represents a heterogeneous group of rare endocrine disorders caused by (epi) genetic abnormalities affecting the GNAS locus. It is mainly characterized by resistance to PTH and TSH, and by peculiar clinical features such as short stature, obesity, cognitive impairment, subcutaneous ossifications and brachydactyly. Delayed puberty, GHRH and calcitonin resistances have also been described. The healthcare-pathway recently proposed by the Italian Society of Pediatric Endocrinology and Diabetology (ISPED) has provided a standardized clinical approach to these conditions. The purpose of the present study was to evaluate its application in clinical practice, and to collect data for setting future specific studies.

**Methods:**

Through a semi-structured survey, based on the indications of the care-pathway, data on PHP clinical management were collected. The compilation of each data in the survey was read as an index of the adoption of the healthcare-pathway in clinical practice.

**Results:**

In addition to the proposing Center, 4 Centers joined the study, thus obtaining a large collection of data on 48 PHP patients. Highest rates in the completion of data were obtained for diagnostic history, auxological measurements and subcutaneous ossifications evaluation. As expected, the availability of data for the other investigated fields was lower, coming from recent research studies. More information has been obtained on hormonal resistance classically involved in PHP (PTH, TSH, GHRH and GnRH) and on cognitive impairment, while a few data has been collected on bone mineral status, calcitonin levels and glucolipid metabolism.

**Conclusions:**

The presented data show that the ISPED healthcare-pathway could represent a valid tool both to confirm the clinical approach to PHP patients and to allow homogeneous data collection; however, it has not yet been fully adopted. The strengthening of the network among the major Italian Endocrine Centers will contribute to improve its application in clinical practice, optimizing the follow-up of these patients and increasing knowledge on PHP.

## Background

The term Pseudohypoparathyroidism (PHP) identifies a heterogeneous group of hereditary disorders caused by (epi) genetic [1] alterations affecting the GNAS locus, on chromosome 20q13 [2, 3] Within this locus, the homonymous gene encodes for Gsα protein, which is involved in the intracellular signal transduction of multiple peptide hormone signals and whose altered function explains the complex clinical manifestations affecting PHP patients [4]. In particular, these disorders are mainly characterized by the resistance to biological actions of the parathyroid hormone, resulting in hypocalcaemia and hyperphosphataemia, in the presence of high PTH levels [5]. Resistance to TSH is also frequent and often represents the first manifestation of the disease, even detected by neonatal screening [6, 7]. Resistance to other multiple hormones such as GHRH [8–10], GnRH [11], calcitonin [12] and insulin [13] is variably observed in PHP patients [14].

The historical classification of PHP differentiated PHP I from PHP II on the basis of the variation in urinary cAMP levels after exogenous administration of synthetic PTH [15, 16], and further distinguished PHP I in type a, b and c [17], on the basis of hormonal resistances and the presence of the Albright Hereditary Osteodistrophy phenotype [18, 19], which includes peculiar physical features such as short stature, early onset obesity, round face, mental retardation, subcutaneous ectopic ossifications and brachydactyly (Table 1). However, subsequent studies have highlighted both a clinical and molecular overlap among the classically described PHP subtypes [20, 21], therefore a new classification has been proposed by an European network of experts on PHP. The nomenclature “Inactivating PTH/PTHrp signalling disorders” (iPPSDs) has been introduced to encompass all the disorders related to this path [22]. The major criteria proposed for clinical diagnosis currently include resistance to PTH, ectopic ossifications and brachydactyly. Minor criteria are represented by resistance to TSH and other hormonal resistance, motor and cognitive impairment, intrauterine and postnatal growth restriction, obesity or overweight and facial dysmorphism. Compared to the previous classification based exclusively on the phenotype, iPPSDs can be further classified from iPPSD1 to iPPSD6 accordingly to the underlying molecular (epi) genetic defect, thus achieving an accurate clinical-molecular definition of each condition (Table 2).

**Table 1:** Historical Classification of PHP and PHP-related disorders

	AHO	Hormone resistance	Heterotopic ossification	PTH test response	***GNAS*** defect
PHP-Ia	Yes	Multiple: PTH, TSH, FSH/LH, GHRH	Yes, superficial	↓cAMP ↓phosphaturia	Maternal inactivating mutations
PPHP	Yes	No	Yes, superficial	Normal	Paternal inactivating mutations
PHP-Ib	No	PTH, TSH	No	↓cAMP ↓phosphaturia	Imprinting dysregulation
PHP-Ic	Yes	Multiple: PTH, TSH, FSH/LH	Yes, superficial	↓cAMP ↓phosphaturia	Maternal inactivating mutations
POH	No	No	Yes, deep	NA	Paternal inactivating mutations

*Abbreviations*: *cAMP* cyclic AMP; GHRH, GH releasing hormone; PTH parathyroid hormone, *TSH* thyroid-stimulating hormone, *FSH/LH* Follicle Stimulating Hormone/Luteinizing Hormone; *POH* progressive osseus heteroplasia

**Table 2:** New Classification and nomenclature of “Inactivating PTH/PTHrP Signalling Disorders” (IPPSDs) – Adapted from Position Statement EuroPHP Network 2016

iPPSD	Molecular Cause	Main Features
iPPSD1	Mutations in the coding sequence of *PTH1R* gene	PTH resistance and/or brachydactyly
iPPSD2	Mutations in the coding sequence of *GNAS* gene (formerly PHP1A, PHP1C, PPHP/AHO/POH)	PTH resistance and/or subcutaneous ossifications and/or brachydactyly
iPPSD3	Abnormal methylation at the *GNAS A/B:*TSS-DMR (formerly PHP1B)	PTH resistance
iPPSD4	Mutations in the coding sequence of *PRKAR1A* gene	PTH resistance and/or brachydactyly
iPPSD5	Mutations in the coding sequence of *PDE4D* gene	Brachydactyly
iPPSD6	Mutations in the coding sequence of *PDE3D* gene	Brachydactyly +/− hypertension

*Abbreviations*: *iPPSDs* Inactivating PTH/PTHrP signaling disorders

In Italy, the great cooperation among Centers that are part of the Group of Study “Endocrine diseases due to Gsα protein impaired function” of the Italian Society of Pediatric Endocrinology and Diabetology (ISPED) has led to the drafting of a shared healthcare-pathway [23]. For rare diseases, healthcare-pathways that derive from the effective and fruitful exchange of experiences among clinicians, represent important tools for the standardization of care among Centers. At the same time, they allow to collect uniform and significant amount of data, essential to further investigation of the less studied clinical-molecular aspects, especially in complex and heterogeneous diseases, such as PHP.

The purpose of the present study was to evaluate the application in clinical practice of the ISPED healthcare-pathway of PHP in the main Italian Centers, to depict the current state of diagnosis and follow up of PHP, outlining any critical issues and providing suggestions for future improvement. Secondary purpose was to collect homogeneous data within the ISPED Study Group “Endocrine diseases due to Gsα protein impaired function”, to set up subsequent multicenter studies on the specific clinical aspects of PHP not yet clarified, aimed at further increasing knowledge about these rare and complex disorders.

## Methods

Based on the ISPED Healthcare-pathway for PHP and following the indications of the International Consensus Statement, a semi-structured survey was conceived. To reach all the Hospitals and University Departments of Pediatrics scattered throughout the country that deal with this rare disease, the survey was sent by e-mail to the clinicians who work in Italian Endocrine Centers participating to the ISPED Study Group “Endocrine diseases due to Gsα protein impaired function”. Clinicians were asked to complete the survey with the available retrospective data of the PHP patients followed between 2010 and 2020 at their Center. After receiving a first preliminary response, two calls were sent to increase the reply from Centers and to encourage further data collection, also highlighting the strength and potential of this multicenter study. The collected data were analyzed after one-year period from the first survey release.

In particular, the required data on pregnancy and delivery history were aimed at exploring the course of pregnancy, gestational age and type of birth, APGAR neonatal score, weight and length. Medical history included age at clinical diagnosis and possibly available molecular confirmation. Auxological parameters (height, weight and target height) at last visit were requested for each patient. Resistance to PTH and its complications were evaluated at diagnosis and at the last control by looking for serum levels of PTH, calcium, phosphorus, alkaline phosphatase, the initial therapy, any changes over time and eventual brain investigation for calcifications. At least one urinary calcium/creatinine ratio measurement and one renal US study were required; the average timing of these evaluations during follow up was also considered, if available. One assessment of mineral bone density by Dual-energy X-ray absorptiometry (DEXA) and/or Quantitative ultrasounds (QUS) was also requested. Thyroid function analysis included TSH and thyroid hormone levels at diagnosis and last control, thyroid ultrasound study during follow up, and replacement therapy. The availability of at least one value of calcitonin was also investigated. Regarding GHRH resistance, the survey examined whether GH secretion had been investigated and whether one IGF1 value was included during follow up; at least one assessment of bone age and the chronological age at which it was performed completed the evaluation. Focusing on GnRH resistance and gonadal function, age and height at the beginning of puberty, basal gonadotropins levels and after stimulation, testosterone values and possible cryptorchidism in males, 17β-estradiol, age at menarche and regularity of periods in females were required.

The evaluation of glycolipid metabolism included data on basal levels and possibly after Oral Glucose Tolerance Test (OGTT) of glucose and insulin, on glycated hemoglobin, total and HDL cholesterol and triglycerides, on blood pressure measurement, on diagnostic or therapeutic interventions for any sleep apneas, as well as information on diet or any treatment and their adherence.

For subcutaneous ossifications, information on number, time of onset, location, related symptoms, evolution over time, any diagnostic investigations or adopted therapies was required.

The age of verbal and motor acquisitions, the need for rehabilitation programs, school or social/psychological support, as well the evaluation of mental functions with objective tests, i.e. IQ (Intelligence Quotient), WISC (Wechsler Intelligence Scale for Children) [24] or WAIS (Wechsler Adult Intelligence Scale) [25] tests were investigated to assess neurocognitive development.

Through a purely descriptive statistical analysis, the percentage of each data collected from the Centers that joined the study was calculated for each of the above listed outcomes. The availability and accuracy in completing the survey were read and evaluated as an index adhesion of the Centers to the PHP healthcare-pathway.

The study was approved by the Local Ethics Committee; in the survey, patients’ data were entered by each clinician anonymously.

## Results

In addition to the proposing Center, other 4 Centers joined the study, allowing to obtain data on a cohort of 48 patients with PHP.

**Fig. 1:**
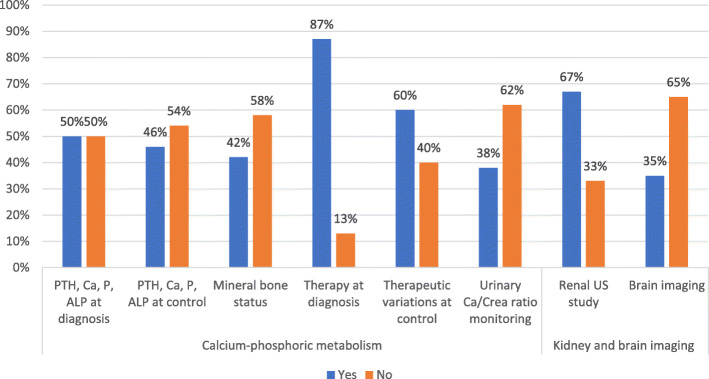
Calcium-phosphorus impairment evaluation

**Fig. 2:**
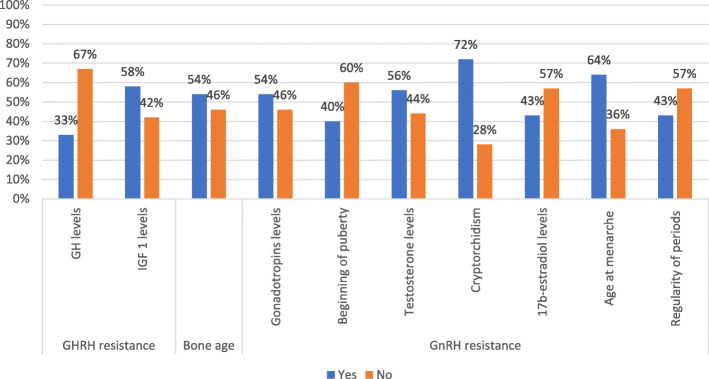
GHRH and GnRH resistance evaluation

**Fig. 3:**
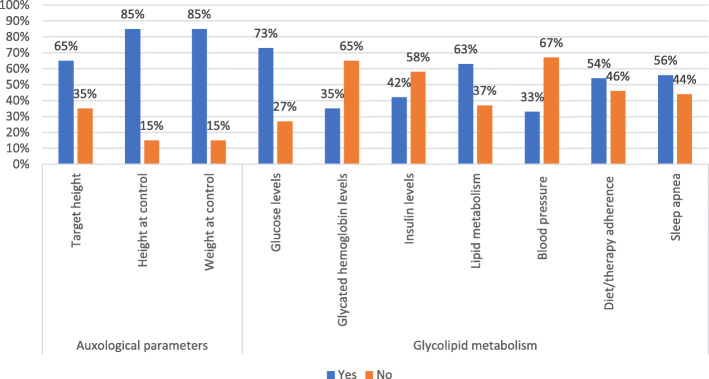
Auxological parameters and glycolipid metabolism

**Fig. 4:**
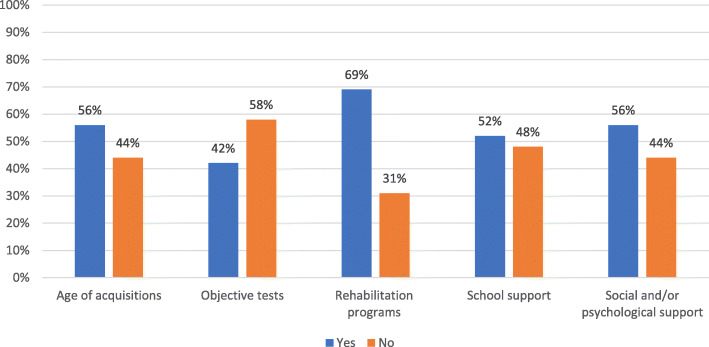
Neurocognitive development

Data collection rates for the main survey items are shown in Table 3. These overall results become more understandable by analyzing the results of the responses of each specific area (Fig. 1, 2, 3, 4).

**Table 3:** Data collection rates for the main items of the survey

Outcomes	Data collection		
	Complete	Partial	Absent
Diagnostic history	69%	19%	12%
Pregnancy and delivery history	21%	54%	25%
PTH resistance and calcium-phosphorus metabolism	17%	79%	4%
Kidney and brain calcifications	36%	58%	6%
TSH resistance and thyroid function	21%	71%	8%
Calcitonin resistance	21%	/	79%
GHRH resistance	31%	44%	25%
GnRH resistance and gonadal function	50%	31%	19%
Auxological parameters	60%	30%	10%
Glycolipid metabolism	19%	69%	12%
Subcutaneous ossifications	69%	17%	14%
Neurocognitive development	27%	54%	19%

Age at clinical diagnosis was indicated in 40 out of 48 patients (83%); mean age at diagnosis was 6.41 years + − 4.51 DS (range 0.17–17 y.o.), while the mean age at study evaluation was 12.92 years + − 5.84 DS (range 2.17–29.11 y.o).

Data on molecular investigations were available for 35 patients of the entire cohort (73%), even if genetic or epigenetic definition was indicated for 22 patients out of these 35 patients (63%): GNAS gene mutations have been reported in 19 (86% PHP-Ia/Ic), metilation GNAS defects in 2 (PHP-Ib 9%) and PDE4D mutations in 1 out of the 22 patients (5%).

Gestational age and type of delivery were reported in 32 and 31 patients (67 and 65%), respectively, but information on the course of pregnancy was not available in nearly half cohort (23/48, 48%); in 30 patients (62%) APGAR score was missing, while the availability of neonatal measurements was discordant, as weight was reported for 36 (75%), while length for 24 (50%) patients. Non physiological pregnancy was described in 8/25 patients (32%), preterms before 37 weeks of pregnancy in 5/32 patients (16%), delivery impairment in 12/31 patients (39%), 7/36 patients were small for gestational age (19%), 2/36 patients were large for gestational age (6%), Apgar score was equal/low than 7 in 4/18 patients (22%).

Focusing on PTH resistance, complete data on calcium-phosphorus metabolism evaluation were recorded in half patients at diagnosis and last control; however, by excluding ALP investigations, these rates increased to 75% (36/48) and 73% (35/48) at diagnosis and last control, respectively. The evaluation of the mineral bone state was missing in over half of the patients (28/48), while at least one urinary calcium/creatinine ratio was available in 30 patients, although monitoring of this parameter was reported only in 18 patients. Interestingly, excluding both measurement of ALP and bone mineral status, the percentage of data completion on calcium-phosphorus metabolism increased from 17 to 50%. Initial therapy was reported in almost all cases, while less information was available on any therapeutic changes at last check-up. Data on renal ultrasound were more available rather than on brain imaging (Fig.1). For calcitonin levels, data were missing for most of the cohort patients (38/48).

As regards resistance to TSH and thyroid function, TSH and FT4 values were collected in 32 patients at diagnosis but in only 16 at the last control; the ultrasound study on the thyroid gland was available for 34 patients. Initial replacement therapy and eventual therapeutic changes at the last control were reported for 37 and 30 patients, respectively.

In the evaluation of GHRH resistance, GH secretion after stimulus was studied in one third of the patients, IGF1 measured in more than half and bone age available for about half patients. For GnRH resistance and gonadal function assessment, hormonal evaluations were reported for almost half patients, cryptorchidism was recorded in the majority of males, while age at menarche and regularity of periods in 9 and 6 out of 14 subjects who had already had menarche, respectively. Age and height at the beginning of puberty were available in 14 out 35 pubertal patients (Fig.2).

Auxological parameters of height and weight were accurately reported in most of cases, whereas data on target height were lacking in almost one third of patients.

Glycolipid metabolism data were fully provided in 9 patients of the total cohort: glucose levels and lipids assessment, was reported in the majority of patients, unlike insulin and glycated hemoglobin levels mostly missing. Blood pressure parameters were available for one third of patients, while data on adherence to diet and obesity treatments or any diagnostic or therapeutic interventions in case of sleep apnea for about half of the patients (Fig. 3).

The study of subcutaneous ossifications was reported for the majority of cohort subjects (85% of cases) and these lesions were detected in 15/48 patients; for all, data on the number, time of onset and location were available, while related symptoms and evolution over time were reported in 10/15 and 7/15 patients, respectively. Any diagnostic investigations and therapies were indicated in 11/15 and in 8/15 cases, respectively.

Finally, to define the neurocognitive development study, data on the age of verbal and motor acquisitions were obtained in 27 patients, and for 20 patients the clinicians specified if objective tests for the assessment of mental functions were performed. The need of rehabilitation programs was indicated for 36 patients, scholastic and social/psychological support for 33 and 27 patients, respectively (Fig.4).

## Discussion

This is a national multicenter study in which the application of the healthcare pathway on PHP issued by Italian Society of Pediatric Endocrinology and Diabetology (ISPED) [23] was investigated. The main Italian Centers of reference for this rare disease participated in the study, with the attempt to define the current status of the diagnostic path and follow-up of patients with PHP in each center, to make it homogeneous among Centers and to investigate which aspects need to be improved. In general, the collected data show that most of the indications of the path on the diagnosis and management of patients with PHP are already part of normal clinical practice, in particular those relating to aspects that have long been known in the literature; for other more recent aspects, greater attention and homogeneity of behavior among Centers is required. For still others, to be considered as a starting point for future researches, the collection of multicenter data will allow to verify their real value for diagnosis or follow up.

The lack of some responses to the survey has to be interpreted as a normal difference among the Centers and a normal updating process of clinical practice not yet completed for this complex disease. The fact that few Centers took part in the survey confirms the low incidence of the disease, whose management remains the prerogative of some centers with experience over time, as occurs for most rare diseases and as indicated by all the main international networks that deal with rare diseases. After the publication of diagnostic-therapeutic pathways, their application to the clinical practice is often not investigated. The real sharing of the ISPED diagnostic-therapeutic path on PHP among expert Italian Centers will facilitate the creation of a homogeneity in the management of the disease, which can subsequently be transferred also to less experienced Centers for homogeneous treatment even near the residence of each patient.

Despite the participation of a few Centers in the study, the survey allowed to collect a large number of data on 48 subjects affected by PHP, which represent a considerable case series, useful for future investigations.

Taking into consideration the different aspects of the disease investigated, it is important to make some clarifications and comments. The collection of data was complete in the majority of patients on only three aspects, namely diagnostic history, auxological measurements and evaluation of subcutaneous ossifications, which represent the first medical actions at the basis of taking charge of a subject with this rare disease and one of the most specific parameters for the clinical suspicion of the disease [14, 22], easy to identify and monitor over time. Noteworthy is the high rate of confirmation of the molecular diagnosis, the importance of which is well known to the reference Centers, for a complete diagnostic definition, more specific follow-up over time and adequate genetic counseling for risk assessment of the recurrence of the disease in the family.

Conversely, data on pregnancy and childbirth were partial or even absent, but it would important to collect them, since to date the only data available concern weight and length at birth, which appear to be different between PHP-Ia and Ib [14]; such data could be regarded as an early diagnostic handle for guiding the most specific molecular analysis.

Although calcium-phosphorus metabolism is probably the most studied aspect in the literature, some new aspects have not yet entered in the normal clinical practice, such as bone mineral density, whose trend is not fully known in PHP subjects [26].

Data on imaging showed that renal ultrasound control has been reserved mainly to patients with persistent hypercalciuria or specific clinical indications, since PHP seems to less associated to kidney calcification than other conditions [14, 23], while brain investigation has been conducted in few patients, as currently indicated, only in case of neurological manifestations [14].

Data on thyroid function were not always present, while it is well known that it should be measured from the first diagnostic suspicion of PHP, as resistance to TSH often represents the first hormonal resistance that appears, already detectable at neonatal screening [7, 26], and should be monitored over time for the correct adjustments of the replacement therapy and to follow its evolution throughout life; as well as the possible resistance to GHRH should be earlier identified, in order to obtain better growth gain with hrGH treatment before the premature closure of the growth cartilages typical of these subjects [8–10, 14].

From the study of calcitonin, interesting data will come on the prevalence and symptoms related to its resistance and the evolution over time, useful to indicate the correct timing for its investigation and monitoring; on the other hand, data collection on GnRH resistance, on the neurocognitive impairment, on glucose-lipid metabolism and blood pressure in a wide serie of PHP subjects will improve their management even after the transition of care to adulthood [13, 27].

Overall, new studies following the extensive application of this common healthcare pathway for the iPPDs could further elucidate differences or overlaps between the different subtypes [14].

## Conclusions

In conclusion, the present study had the main purpose of investigating the clinical application to the national care-pathway indications. However, thanks to the deep collaboration among centers within the ISPED Group of Study, huge amount of clinical data have been obtained for each specific aspect of PHP, useful for future multicenter studies. Thus, for rare diseases, the creation and the adoption of a shared care pathway could have positive effects not only in improving patients’ assistance, but also in increasing the disease knowledge.

## Data Availability

The datasets used and/or analysed during the current study are available from the corresponding author on reasonable request.

## Abbreviations

PHP: Pseudohypoparathyroidism
iPPSDs: Inactivating PTH/PTHrp signalling disorders
ISPED: Italian Society of Pediatric Endocrinology and Diabetology
DEXA: *Dual*-energy *X*-*ray absorptiometry*
QUS: *Quantitative* ultrasounds
OGTT: Oral Glucose Tolerance Test
IQ: Intelligence Quotient
WISC: Wechsler Intelligence Scale for Children
WAIS: Wechsler Adult Intelligence Scale
AHO: Albright hereditary osteodystrophy
